# Trajet aberrant de l'artère carotide interne

**DOI:** 10.11604/pamj.2015.22.257.8102

**Published:** 2015-11-18

**Authors:** Rim Lahiani, Madiha Mahfoudhi

**Affiliations:** 1 Service ORL, Hôpital Charles Nicolle, Tunis, Tunisie; 2 Service de MédecineInterne A, Hôpital Charles Nicolle, Tunis, Tunisie

**Keywords:** Artère carotide interne, oropharynx, scanner cervical, artery internal carotid, oropharynx, cervical scanner

## Image en medicine

L'artère carotide interne aberrante est une malformation congénitale rare. Les malpositions de cette artère sont bien mises en évidence sur la TDM injectée ou mieux sur l'IRM (avec des séquences angiographiques) qui est considérée l'examen le moins invasif et permettant de confirmer l'anomalie et les rapports de l'artère carotide interne avec les autres structures du pharynx. Par son siège vulnérable, cette artère, au contact direct de la muqueuse de la tonsille palatine, représente un risque fatal lors d'un geste chirurgical même anodin (amygdalectomie, véloplastie, adénoïdectomie) ou un geste invasif de la région pharyngée. Les précautions à prendre avant tout geste chirurgical impose une palpation systématique de la région pharyngée. Toute asymétrie oropharyngée doit inciter à demander une imagerie. Patiente âgée de 55 ans, sans antécédents pathologiques particuliers, a consulté pour une odynophagie sans fièvre ni altération de l’état général. L'examen physique a objectivé un bombement du mur postéro-latéral droit de l'oropharynx refoulant l'amygdale droite, avec une muqueuse normale en regard. Le reste de l'examen était sans anomalies. L'examen biologique n'a pas noté d'anomalies. Plusieurs diagnostics ont été évoqués en particulier un lymphome ou une néoplasie solide. Un scanner cervical injecté a objectivé un trajet aberrant de l'artère carotide interne dans sa portion cervicale, décrivant une boucle sous muqueuse, refoulant le mur postéro-latéral droit de l'oropharynx. Le diagnostic d'un trajet aberrant de l'artère carotide interne a été alors posé.

**Figure 1:**
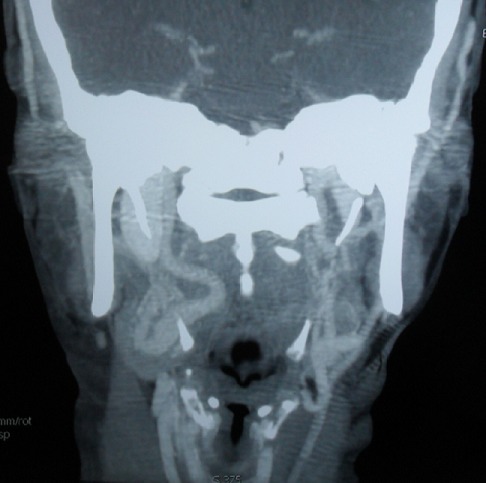
TDM injectée à l’étage cervical en coupe coronale: boucles sous muqueuse de l'artère carotide interne

